# Variation in the Association of Body Types to Decreased High-Density Lipoprotein in Older Adults (NHANES 2005-2014)

**DOI:** 10.1093/geroni/igaa057.688

**Published:** 2020-12-16

**Authors:** Queendaleen Chukwurah

**Affiliations:** Tulane University, New Orleans, Louisiana, United States

## Abstract

General obesity and central obesity represent cardiovascular disease risk factors and are known to be related to dyslipidemia. I examine the variation in the association of combined body mass index/waist circumference classification to decreased high-density lipoprotein cholesterol (HDL-C). Body mass index /waist circumference (WC) cut off values were used to create six body types: normal weight with normal WC (NWT-NWC), overweight with normal WC (OWT-NWC), obese with normal WC (O-NWC), normal weight with high WC (NWT-HWC), overweight with high WC (OWT-HWC), and obese with high WC (O-HWC). HDL-C was defined as decreased if < 40 mg/dl for men or < 50 mg/dl for women and normal if ≥ 40 mg/dL for men or ≥ 50 mg/dL for women. Sample population included 5,772 participants of the National Health and Nutrition Examination Survey (NHANES 2005-2014) aged 50 years and older. The mean (SD) age was 61.8 (0.2), and 50.5% were females, while 10% were minority. The prevalence of decreased HDL-C was 29.1%. Analysis involved weighted multivariable logistic regression adjusted for age, race-ethnicity, gender, education, poverty-income-ratio, smoking, and alcohol intake. Regression reveals a higher likelihood of decreased HDL-C for OWT-NWC (aOR 2.12 95% CI 1.43,3.15 ), NWT-HWC (aOR 2.57 95% CI 1.59,4.16 ), OWT-HWC(aOR 3.09 95% CI 2.29,4.15 ), and O-HWC (aOR 5.30 95% CI 4.01,6.86 ) when compared to NWT-NWC. These associations are important to public health practice and policies as it demonstrates the implications of the parallel use of anthropometric measures for all body weights in health-risk assessments of older adults.

